# Patient experiences of waiting for orthopaedic care and priorities for ‘waiting well’: a qualitative study in a London NHS trust

**DOI:** 10.1186/s13690-025-01578-4

**Published:** 2025-04-07

**Authors:** Jodie Chan, Sharon Poon, Anna Lawrence-Jones, Fiona O’Driscoll, Carol Waugh, Abiola Awojobi-Johnson, Leila Shepherd, Kate Grailey

**Affiliations:** 1 Helix Centre, Institute of Global Health Innovation, Imperial College London, QEQM Building, St Mary’s Hospital, Room 1035, London, W2 1 NY UK; 2 Imperial College Healthcare NHS Trust, London, UK

**Keywords:** Waiting lists, Orthopaedics, Health equity, Deprivation, Underserved groups, Public involvement, Co-design

## Abstract

**Background:**

Following the COVID- 19 pandemic, patients are facing larger waiting lists and longer waiting times than ever before. Long waits for orthopaedic treatment can negatively impact patients’ quality of life due to pain, reduced mobility, and the psychological effects of waiting itself. Initial analysis at a London National Health Service (NHS) Trust showed that patients living in the most deprived areas were more likely to face longer waiting times for joint replacement surgery. This study aimed to understand what would support people to live well while waiting for orthopaedic treatment, focusing on those in the most deprived areas.

**Methods:**

We conducted semi-structured interviews with patients living in deprived areas in North West London who were currently waiting or had recently waited over 18 weeks for an orthopaedic procedure. Data were analysed using inductive thematic analysis. Key insights from this analysis were brought to a co-design workshop, where patients developed targeted and bespoke support initiatives and identified how these might help people to live well while waiting. We worked with two public partners throughout, who shaped the interview questions, analysis, and workshop.

**Results:**

Seven patients were interviewed, and a further six patients participated in the co-design workshop. The interviews identified four immediate impacts of waiting on wellbeing, including physical pain, limitations on daily activities, greater reliance on friends and family, and anxiety around the wait; as well as four long-term consequences of an extended wait time, including physical deterioration, delays of life plans, changes in hopefulness, and reduced trust in the NHS. Additionally, they identified four sub-themes around patients’ support needs while waiting: medical support, practical support, informational support, and emotional support. Based on these thematic insights, patients at the workshop developed four potential interventions to support people waiting for orthopaedic treatment.

**Conclusion:**

This study highlights the negative impacts that prolonged waits for orthopaedic treatment can have on patients’ physical, mental, and social wellbeing, and notes that patients with caring or financial responsibilities may be disproportionately affected. The support needs expressed by patients focused more on acknowledgement of their concerns and management of their expectations by health professionals, rather than on advice around lifestyle changes and coping mechanisms. Our study offers a number of support ideas proposed by patients which can be further developed and implemented by health services to better support patients to live well while waiting for orthopaedic treatment.

**Supplementary Information:**

The online version contains supplementary material available at 10.1186/s13690-025-01578-4.

**Table:** 

Text box 1. Contributions to the literature	
• Patients are facing longer waits than ever before, but there is limited research on the impacts of waiting for elective surgical care on patients’ wellbeing and quality of life. • Patients identified significant impacts from extended waits, including physical deterioration, delays of life plans, changes in hopefulness, and reduced trust in the NHS. • While health systems often focus on prehabilitation, patients were more interested in acknowledgement of their concerns and management of their expectations by health professionals. • This research explores what ‘waiting well’ means from a patient perspective and offers patient-developed ideas for how health services can support this.	

## Background

More patients are waiting for hospital care in the United Kingdom (UK) than ever before. Having already reached 4.6 million by early 2020, the National Health Service (NHS) waiting list grew sharply when non-urgent elective care was suspended to deal with the COVID- 19 pandemic [2]. As of June 2024, the NHS waiting list for elective treatment in England was 7.6 million [2]. Within that, trauma and orthopaedics had the largest waiting list, with more than 800,000 people waiting for treatment [22].

The UK is not alone in this – virtually all European health systems experienced some level of disruption as a result of the COVID- 19 pandemic [15, 18, 23]. Because of the elective care missed during this time, it is not enough for countries to simply restore service provision to pre-pandemic levels; this only prevents waiting lists from growing further [15, 23]. Instead, health systems must increase care provision to above previous levels to overcome the backlogs that have built up. For countries such as the United Kingdom, which already faced large waiting lists before the pandemic, this is a significant challenge [15, 18, 23].

One consequence of larger waiting lists is an increased time to treatment for patients. In 2012, NHS England set a standard stating that at least 92% of patients should start treatment within 18 weeks of referral [12]. However, this standard was last met in 2015, and currently only 57% of patients wait less than 18 weeks for treatment [7, 22]. Waiting longer for treatment puts patients at increased risk of experiencing the negative consequences of their medical condition, such as pain and reduced mobility, and a patient’s condition may worsen over time [6, 8, 24]. This can result in poorer quality of life and a greater chance of social isolation [17, 24]. Additionally, the wait itself can significantly impact patients’ mental wellbeing, leading to increased symptoms of anxiety, depression, and stress [6, 17, 24].

Waiting for treatment is experienced differently by different individuals, and the impact of waiting may worsen health outcomes and wellbeing more so for some patient groups than others, particularly if these groups are waiting longer. One scoping review found that waiting had a greater impact on mental health for patients of lower socio-economic status, as well as women, new immigrants, and younger patients [4]. People living in more deprived areas are more likely to have multiple long-term conditions, have their condition deteriorate more quickly, and develop complications while waiting [19]. Waiting for treatment can also be more disruptive for those with caring or financial responsibilities. A 2022 Healthwatch survey found that waiting negatively impacted the ability to care for others for 31% of those on lower incomes, compared to 20% of those on higher incomes,similarly, waiting impacted the ability to work for 39% of those on lower incomes, compared to 29% of those on higher incomes [5, 10].

In the elective care recovery plan published in February 2022, NHS England asked NHS sites to work on inclusive recovery by identifying inequalities within waiting lists relating to deprivation and ethnicity, and then prioritising service delivery by taking this into account [13]. In response, one London NHS trust analysed data on waiting times for orthopaedic procedures, looking for evidence of differences in waiting times according to protected characteristics. Initial analysis showed a notable difference in waiting times for joint replacement surgery according to Index of Multiple Deprivation (IMD). This is consistent with national analysis by the King’s Fund, which found that people in the most deprived areas were 2.1 times more likely to wait over a year for elective care compared to people in the least deprived areas [19].

Longer waiting times among those living in more deprived areas could be due to a number of reasons. People in more deprived areas may face more practical barriers to attending appointments, such as difficulties getting time off work, managing caring responsibilities, or arranging transport, and missed appointments can lead to delays in treatment [19, 21]. Unclear or insufficient communication regarding appointments can also lead to delays, particularly for people whose preferred language is not English, those with low digital literacy, and those with less trust in the health system [19, 21]. Healthwatch found that only 44% of people with lower wealth were given a clear NHS point of contact, compared to 55% of those with higher wealth [5, 10].

While vital work is ongoing to increase elective care provision and overcome the NHS backlogs, it is also important that we provide support for the millions of patients currently waiting for treatment. This study aimed to understand, from patients, what would support people to live well while waiting for orthopaedic treatment, focusing on patients living in deprived areas who are more likely to face longer waits.

In particular, the study aimed to:

1. Understand the experiences of patients living in more deprived areas while waiting for orthopaedic treatment.
2. Co-design targeted and bespoke support initiatives and identify how these might help people to live well while waiting.

For this study, living well was defined as having a good quality of life while waiting, rather than focusing on specific lifestyle changes made in preparation for surgery.

## Methods

### Study design

This project was undertaken as a qualitative service evaluation and received the relevant approvals from the clinical governance team at the study site (reference number 980). This was an exploratory study which used semi-structured interviews and an inductive thematic analysis approach to understand the experiences of patients living in more deprived areas while waiting for orthopaedic treatment. An inductive approach was selected as the study team did not have pre-conceived ideas regarding the themes present in the data. Thematic analysis was selected in line with Braun & Clarke’s methodology as it was felt this was the best approach to understand the qualitative data, but that was also accessible for public members involved in the analysis [1]. Following thematic analysis of the interview data, a workshop was held with patients to co-design targeted and bespoke support initiatives, informed by the interview insights, to help people live well while waiting. The study is reported in line with the Standards for Reporting Qualitative Research [14].

### Study setting

This study took place at a large NHS Trust in London and focused on their orthopaedic service. Orthopaedics was chosen due to the high volume of patients and the initial analysis showing a disparity in waiting times by deprivation.

### Patient and public involvement

To ensure this project was informed by those with lived experience, we recruited two public partners with lived experience of waiting for orthopaedic treatment to work with us throughout the project. Public partners were involved in reviewing recruitment materials, developing the interview discussion guide, coding interview transcripts, taking part in the thematic analysis, designing the workshop format, supporting patients to participate in the workshop, and reviewing the insights report.

### Sampling and recruitment

We used purposive sampling to recruit patients who varied by ethnicity, gender, age, and reason for procedure and who met the following inclusion criteria:

- Living in a more deprived area (IMD quintile 1), AND
- Currently waiting for an orthopaedics procedure for over 18 weeks, OR
- Previously received an orthopaedics procedure in 2023 after waiting for over 18 weeks.

Eligible patients were identified using data provided by the study site’s Business Intelligence (BI) team. Patients currently on the waiting list were prioritised for interviews, as their experience of waiting was more current, may be easier to recall, and would not be influenced by their treatment outcome.

Selected patients were contacted by phone and invited to take part in the study, either by doing an interview at a time that was convenient for them or by taking part in the workshop. The target sample size was eight patients for the interviews and six for the workshop. This was determined based on the total number of eligible patients (the BI team identified 189 patients who met the inclusion criteria), an expectation informed by previous work that the proportion of patients agreeing to take part would be low due to competing responsibilities and mistrust of research and the health system, and project resource and time constraints. The goal was to achieve thematic saturation, but it was acknowledged from the outset that this may not be possible given the resource available and potential challenges with recruitment. There was no overlap between the patients who took part in an interview and those who took part in the workshop. Prior to phoning, a text message was sent by the orthopaedics team to let patients know to expect a call from the study site. Interpreters were available for patients whose preferred language was not English.

### Data collection - interviews

All patient interviews took place in March 2024 and were conducted by JC. A second member of staff (ALJ, FO) was present at each interview to transcribe notes verbatim. Interviews were also recorded and transcribed through Microsoft Teams. Written informed consent was gained prior to carrying out the interviews. Interviews were planned to last up to one hour and were carried out over the phone, on a video call, or in-person at the research team’s hospital location, depending on the patient’s preference.

The semi-structured interview guide (Additional file 1) was developed based on discussions with clinical staff (CW) and public partners (AAJ) and covered the impact of waiting on the patient; any coping strategies used while waiting; any support or communication received while waiting; and ideas for how to improve the experience. One public partner suggested including a question on the impact of waiting on patients’ relationships with their family and friends; this had not been previously considered by the research or clinical teams and was included in the guide. Interview findings were used to iterate the discussion guides during the data collection period.

### Data analysis - interviews

We used an inductive thematic analysis approach to analyse our interview data. Thematic analysis was chosen as it is relatively quick and easy to learn, and was therefore accessible for our public partners (AAJ) to get involved in. Additionally, the flexibility of this approach allowed for unanticipated insights to emerge.

Our thematic analysis process followed a six-phase approach, comprising of familiarisation with the dataset, generation of codes, construction of themes by collating codes, and then review, finalisation, and naming of themes [1]. All patient interview transcripts were reviewed by one researcher to become familiar with the dataset and generate codes (JC). Transcripts were then analysed by a second team member – either a researcher (FO, SP) or a public partner (AAJ) – and compared with the initial analysis. Public partners received training on qualitative analysis and reflexivity before reviewing the transcripts. Most of the codes generated were consistent between team members, with any discrepancies discussed to reach agreement on inclusion or removal. A framework with themes and sub-themes was developed through discussion, review, and iteration by the researcher (JC), clinical partner (CW), and public partners (AAJ).

### Data collection - workshop

The workshop took place in April 2024 at a community venue and lasted two hours. The workshop was facilitated by JC and ALJ, with two additional members of staff present to take notes (CW). The public partners (AAJ) contributed to activities and provided support and encouragement to participants throughout the workshop. A Farsi interpreter was also present to support the participation of one patient. Written informed consent was gained prior to the workshop.

The goal of the workshop was to co-design possible initiatives to support patients to live well while waiting, building on the identified themes from the interviews. To facilitate this, we shared a pre-read with key interview insights ahead of the workshop. This pre-read was translated into Farsi for one participant. At the workshop, the project was introduced, including an overview of the interview insights. Participants then split into two groups and worked together to design possible support initiatives. This discussion was guided by a worksheet (Additional file 2) which was reviewed by public partners and adapted from previous co-design workshops run by the research team.

### Researcher characteristics and reflexivity

Our multidisciplinary research team combined research, clinical, and lived expertise. JC and ALJ are public involvement professionals, FO is a health policy fellow, KG is a postgraduate qualitative researcher with a clinical background in anaesthesia, CW is an advanced physiotherapy practitioner, and AAJ is a patient with lived experience of waiting for an orthopaedics procedure. JC and ALJ have previous experience involving underserved groups in research. FO and KG have previous experience with the conduct and analysis of qualitative studies in the clinical environment.

The team held a discussion around reflexivity prior to coding transcripts. Each member of the team brought different perspectives and experiences, allowing us to consider varied interpretations of the data. Any disagreements were discussed as a group before reaching consensus, minimising the risk that our themes were influenced by the pre-existing biases and understandings of a single researcher.

## Results

### Understanding the experiences of patients living in more deprived areas while waiting for orthopaedic treatment

#### Patient demographics

Seven patients were interviewed for this study. This fell short of the target sample of eight due to challenges with recruitment. One hundred eighty-nine eligible patients were identified by the BI team, and over half were invited to take part in an interview. Fourteen patients accepted the invitation and were scheduled for an interview, but seven of these patients did not attend or cancelled and chose not to reschedule. A decision was made at this point not to invite any further patients to interview in order to ensure there were enough patients left to invite to the workshop. While we did not reach thematic saturation, strong themes emerged through our analysis, and we feel confident to present them as exploratory findings.

All patients interviewed lived in an area with the highest level of deprivation (IMD quintile 1). Four of the patients interviewed were currently waiting for treatment, and three had already received treatment. Further detail on the demographics of the patients interviewed can be found in Table 1. Interviews lasted 30 to 60 min and took place over the phone (*n* = 3), on Microsoft Teams (*n* = 1), or in person at the research team’s hospital location (*n* = 3).

**Table 1:** Participant characteristics for patient interviews

*Category*	*n*	*%*
**Ethnicity**		
Black African	1	14
Black Caribbean	1	14
Black – Any other Black	1	14
Mixed – White and Black African	1	14
White British	2	28
White – Any other White	1	14
**Age**		
20–29 years old	1	14
30–39 years old	1	14
40–49 years old	1	14
60–69 years old	3	43
70–79 years old	1	14
**Gender**		
Female	2	29
Male	5	71
**Reason for procedure**		
Arthritis/age-related condition	5	71
Accident/injury	2	29
**Wait status**		
Currently waiting	4	57
Already treated	3	43

#### Thematic analysis

Thematic analysis of patient interview data led to the development of three high-level themes, each with several sub-themes. The lead researcher (JC) met with the clinical partner (CW) and public partners (AAJ) to review the generated codes and construct the thematic framework shown in Table 2. The first theme identifies the immediate impacts of waiting on patients’ wellbeing, including physical pain, limitations on daily activities, greater reliance on friends and family, and anxiety around the wait. The second theme explores the long-term consequences of an extended wait time, including physical deterioration, delays of life plans, changes in hopefulness, and reduced trust in the NHS. The third theme reflects the increased support needs described by participants as a consequence of waiting: medical support to assist with pain and deterioration; practical support to assist with daily tasks; informational support to ease anxiety while waiting; and emotional support to aid positivity. There was consideration of a fourth theme around protective factors, but the group ultimately agreed this was encapsulated by the other themes.

**Table 2:** Thematic framework for analysis

Theme	Subtheme	Codes
Immediate impacts of waiting	Physical pain	Chronic pain
		Lack of pain
		Increased pain tolerance
	Limitations on daily activities	Restraints on daily tasks
		Reduced social activities
		Reduced exercise
		Limitations on work
		Limitations on caring responsibilities
	Greater reliance on family and friends	Support from family and friends
		Appreciation for family and friends
		Lack of understanding from family and friends
		Frustration with family and friends
		Negative impact on pride
	Anxiety around the wait	Uncertainty of wait status
		Lack of communication
		Fear of being forgotten
		Patience and faith in NHS
		Relief at receiving appointment date
Long-term consequences of extended waits	Physical deterioration	Deterioration
		Fear of additional operations
		Compensation injuries
	Delays of life plans	Delay of major life events due to limited mobility while waiting
		Inability to schedule life events due to anticipation of appointment date
		Mourning of time lost
		Negative impact of false reassurance
	Changes in hopefulness	Patience and faith in NHS
		Loss of hope
		Anger and frustration
		Devastation from last-minute cancellations
	Reduced trust in the NHS	Recognition of pressures on NHS
		Appreciation of and praise for NHS
		Lack of trust in NHS
		Fears of being abandoned
		Concerns around quality of care
		Desire for private care
Support needs as a consequence of waiting	Medical support to assist with pain and deterioration	Negative side effects of pain killers
		Fears of addiction to pain killers
		Diminishing effect of pain killers
		Lack of physiotherapy offer
		Physical inability to do physiotherapy
		Positive impacts of physiotherapy
		Lack of medical options other than operation
	Practical support to assist with daily tasks	Support from family and friends
		Appreciation for family and friends
		Lack of understanding from family and friends
		Frustration with family and friends
	Informational support to ease anxiety	Desire for information around wait time
		Desire for information around operation
		Desire for information around recovery
		Poor communication from NHS
		Difficulty contacting service for support
		Communication preferences
	Emotional support to aid positivity	Encouragement from family and friends
		Reassurance from faith and religion
		Positive impact of informal peer support
		Positive impact of long-term relationships with health professionals

#### Theme 1: Immediate impacts of waiting

The first theme contained data around the immediate impacts on patients’ wellbeing while waiting. This included four sub-themes: chronic pain, limited ability to carry out daily activities, greater reliance on friends and family, and stress from the uncertainty.

Patients’ experience of pain varied. For some, it was a core part of their experience and had a strong negative impact on their wellbeing.

*“I’m in a lot of pain. Arthritis, multiple joint pain. The physical pain is getting worse.” – Black African woman, aged 40–49*

Others felt they got used to the pain or were not experiencing much pain to begin with. Those who had previously received a joint replacement on one side of their body and were now waiting for their second replacement on the other side were generally in less pain after their first operation.

*“You get used to the pain. It’s really weird, your pain threshold gets stronger and stronger. Before the operation, I got used to it.” – Black man, aged 60–69*

All patients shared that their ability to carry out daily tasks was limited while they were waiting for their operation. This included activities such as carrying items, doing the shopping, going up stairs, walking long distances, and doing sports. Some patients also said that they did not feel fit enough to make the lifestyle changes recommended prior to their operation, such as doing more exercise.

*“It has severe restraints. For example, it will take me a few days to recover from walking here to the interview. I’ve got to the stage where I stay indoors and play with laptops and try not to do too much.” – White British man, aged 60–69.*

The loss of mobility was particularly impactful for those of working age who were not able to work while waiting for their operation. It was also impactful for people with caring responsibilities, who had to balance caring for themselves and caring for others. People who lived on their own were also more greatly impacted by limitations in their ability to carry out daily tasks.

*“I have my son who is 17 and born premature. He is physically disabled. Not that I have neglected looking after myself, but I have found it hard. At the moment, I can tell him much about me, as I need to take care of him. It’s painful.” – Black African woman, aged 40–49.*

Linked to their limited mobility, patients often relied on their family and friends for support with daily tasks such as shopping for groceries, picking up medications, and travelling to appointments. This loss of independence impacted patients’ relationships with those close to them, both positively and negatively.

Many patients showed appreciation for their support system and shared that they grew closer to their loved ones as a result of the support provided to them.

*“My friends and family have been very supportive and caring for me. They understand. They have supported me with money, food – I used to work. My mum does a lot for me – cooking and washing. I can’t carry things from one room to another. I have a nice little support network.” – Black Caribbean man, aged 30–39.*

Some felt their pride prevented them from asking for help and said this could be frustrating for friends and family who were trying to offer support.

*“I don’t like asking for help – this is a big thing for me. I don’t know, I feel a sense of embarrassment, shame, [wondering] if people are going to judge. [My friends] say, ‘You can’t do it, doing the dishes or taking the rubbish. You have to stop doing those things.’ I don’t like being told, ‘You need to stop, let me help you.’” – Black African woman, aged 40–49.*

Others asked for help but found that their friends and family did not understand their situation or dismissed their pain. They felt frustration over having to explain themselves repeatedly and this led to strained relationships, causing some patients to isolate themselves.

*“I asked a friend to go to the pharmacy to get my prescription and he’s like, ‘I can’t keep doing this, being like your grandma.’ But he’s the same friend that’s seen me learn how to walk again. He should understand my situation.” – Mixed (White and Black African) man, aged 20–29.*

Several patients reported feeling stress or anxiety during their wait. This was typically around the uncertainty of waiting, rather than worries about the operation itself.

*“I’ve tried to stay relaxed, but there had to be a bit of wear and tear. Hopefully not in mental decline, but background stress due to waiting.”*

These worries were heightened by a lack of communication around where they stood on the waiting list, and several patients shared fears of being forgotten.

*“It might be useful every three months for someone to say how far up on the waiting list you are, without you having to phone. If they sent a letter or phoned me up to say you’re being dealt with in a month or two months. Then you know you are not being forgotten.” – White British woman, aged 60–69.*

Patients generally felt that this anxiety subsided once they had a confirmed appointment date, and preparing for their appointment helped return a sense of control.

*“Once you’ve got a date, I think it’s alright. Once it ain’t six months down the line.” – Black man, aged 60–69*

#### Theme 2: Long-term consequences of extended waits

The second theme explored the longer-term consequences which arose as a result of extended wait times and repeated cancellations. This included four sub-themes: physical deterioration and compensation injuries, delays of major life plans, fears of cancellations, and reduced trust in the NHS.

Those experiencing a lot of physical pain often felt that the length of their wait allowed for their condition to deteriorate, and this was accompanied by fears that their planned operation would no longer be suitable.

*“It’s not like, ‘Oh dear, you need your knee replaced’ and the damage to your knee stops at that point. Over the last three or four months, [my knee] has gone down another step. So now it is very uncomfortable. Before it was tolerable. The background fear is that there is an increased level of damage. The scans the healthcare professionals have are 18 months old. The damage is more extensive than it was. I wouldn’t be surprised if now it’s a whole knee replacement.” – White British man, aged 60–69.*

Some patients also reported pain in other areas of their body after overusing their joints to compensate for the original joint pain, and worried that they would need additional operations as a result.

*“All the years, the other knee was compensating for it. I don’t want to get to the stage where I need the left knee done.” – Black man, aged 60–69*

As a result of their limited mobility, several patients spoke about delaying major life plans and reported a sense of loss over the time wasted while waiting.

*“I don’t know if I might have been doing other things. You can only mourn what you know you have lost. That is the bigger loss. I am running out of years. To have an extra half year would be great.” – White British man, aged 60–69.*

*“I could resume living my life a long time ago – if everything had just been done in the time that it said it would be done. I’ve pushed plans back. I’m planning to get married – I can’t walk down the aisle without an aid. I can’t have a child until this is resolved because there’s a chance I could trip over something and harm my baby. I can’t even talk right now, I’m overwhelmed.” – Mixed (White and Black African) man, aged 20–29.*

This was made worse by false reassurances from hospital staff that the operation would happen soon, as this would lead patients to put their lives on hold in anticipation of an appointment date rather than making plans around what was possible while waiting.

*“When I had my operation planned for 2021, I had a holiday that I had to cancel [because I was worried the appointment would get scheduled while I was away]. That’s happened a few times – I’ve missed out on a good time, a holiday. That’s why I just want them to be honest with me.”*

For some of the patients we spoke to, the stress around their wait was manageable as they felt the operation would ‘come when it comes’ and felt at peace with that. These patients typically noted the current pressures on the health system and showed great appreciation for the performance of the NHS given the circumstances.

*“I know there’s a lot of pressure, all sorts of strikes and everything. It is what it is, got to wait for it. I’m a pretty patient person anyway. Weren’t a problem with me.” – Black man, aged 60–69.*

Others were more greatly impacted by the stresses of waiting and felt that their sense of hope declined as their operation date continued to be pushed back.

*“I feel bloody angry that I have waited this long. My hope in thinking that I am going to be okay has gone down. No one can do anything. There’s no magic solution.” – Black African woman, aged 40–49.*

One patient, who had suffered extensive injury to his leg following an accident, lost all hope of recovery and requested an amputation before being convinced by his family and consultant to wait a bit longer.

*“This is it – this is me for the rest of my life. It was the same story for two years. The third year I said, ‘I’m ready for it. Just cut my leg off. But [my family] said, ‘No, there’s more you can do.” – Mixed (White and Black African) man, aged 20–29.*

The cancellation of appointments also had an acute impact on patients’ wellbeing and sense of hope. Patients noted the mental and physical preparation they did leading up to their operation, particularly after having waited so long for it, and shared the devastation they felt when the operation was cancelled last minute. This happened multiple times for some patients, and they reported a persistent anxiety that the phone would ring with yet another cancellation.

*“Sometimes [the surgery] was cancelled a month in advance, sometimes it was a day before the appointment. I felt like I’d been hit by a lorry again. I’d put things in place, I’d mentally put myself in place. If they are going to cancel, I need to know a week in advance, not the day before. Otherwise, it’s a complete soul crusher.” – Mixed (White and Black African) man, aged 20–29.*

The majority of patients we spoke to did not feel that their view of the NHS changed as a result of their wait. While they expressed frustration with their situation, they recognised the challenges faced by the system and were appreciative of the care delivered amidst these circumstances. Their trust in the NHS helped these patients feel more reassured about their wait and their operation.

*“I still think they do a fantastic job in a difficult situation. I’ve only ever had a lot of help and support from them.” – White British woman, aged 60–69*

However, one patient felt that their trust in the NHS had been negatively impacted by the long wait and poor communication, and this significantly increased their fear leading up to their operation.

*“They need to be more clear. They’ve lied. They could have said we would like to book you in 12 weeks, but realistically it will be within 12 months. The expectation would have been a lot lower. Can I trust that there are going to be appropriate appointments? It doesn’t give you a sense of wellbeing and trust for the NHS. I am petrified they are going to leave me.” – Black African woman, aged 40–49.*

#### Theme 3: Support needs while waiting

The third theme that came out of our analysis reflected the support needs which arose while waiting. This theme contained patients’ reflections on the support they received while waiting, as well as the support they wished they had received. Four types of support were identified: medical support to help with pain and deterioration, practical support to assist with daily tasks, information support to help patients understand what to expect and ease anxiety, and emotional support to help patients stay positive. We also found this support typically came from three sources: health professionals, family and friends, or peers who have been in similar situations.

Regarding medical support, most patients wanted to avoid painkillers where possible because of side effects such as drowsiness and constipation, as well as fears of addiction and dependence. Painkillers were viewed as a short-term solution at best, and several patients preferred to use them for the pain after the operation, rather than during the wait.

*“The painkillers are strong. I am like a zombie if I take them in the day. I can’t manage unless I am at home and have someone to take care of me. Literally, I can’t move around. Short term, the painkillers work okay, but this has been since last year. I don’t want to increase the dose.” – Black African woman, aged 40–49.*

Some patients found physiotherapy useful to help maintain mobility and reduce their recovery time. Patients who had seen firsthand the positive experiences of other patients who had completed their physiotherapy, or the negative experiences of those who had not, were particularly motivated to do their exercises. Others shared that they could not do physiotherapy exercises, or get to the physiotherapy clinic, because of their pain.

*“I think the physio has helped, I must admit, as it keeps you mobile. [What motivated me was] the idea that physio would help in the long run, that my recovery time would be quicker. There are two ladies in our church. The lady with the hip replacement didn’t feel she wanted or needed to do her exercises, and, in a year, she was in a wheelchair. The lady with the knee replacement did all her exercises and now is a very spritely 90-year-old. It showed that doing the exercises works.” – White British woman, aged 60–69.*

Besides painkillers and physiotherapy, patients often felt that there was not much that health professionals could do to support them other than carry out the operation.

As mentioned previously, limited mobility meant that patients often needed practical support with daily tasks such as shopping and transport. Because the NHS does not provide such support, patients often looked to family and friends for help. Some patients had support systems who happily took on this role.

*“[My friends and family] always [supported me] if I needed them. All sorts of things, like shopping or needing a lift. I couldn’t drive. I’ve got friends who are taxi drivers and got cars, [they] could pick me up and drop me off.” – Black man, aged 60–69.*

However, others found that friends and family did not understand their situations and were unwilling to provide the support needed, leaving patients feeling frustrated and on their own.

*“When I say I need to go shopping, sometimes they have let me down, because they expect me to go to the shops and then offer to meet me after. I remind them to be more supportive. When I have reminded them, the conversation hasn’t gone well, and they have been dismissive.” – Black African woman, aged 40–49.*

Patients showed a desire for information around their wait time, operation, and recovery. This information helped patients understand what to expect and eased anxiety while waiting.

Regarding their wait time, patients showed an understanding of the pressures faced by the NHS and identified contributors to their wait time such as a lack of funding, strikes, and the COVID- 19 pandemic. However, many wished the study site had been more upfront about their expected wait time and provided periodical updates on their wait status so that they could plan their lives accordingly. Patients also found it difficult to get through to the right person when they had questions and expressed a desire for a direct line of communication with the orthopaedics service for information and support.

*“I was told that it would be this year, then the year would end, then it would be early next year. I kept waiting for four years, being led like a goose chase. Just be honest with the timing, just tell us. The last time I saw my orthopaedic doctor was last year, and I asked him frankly would this be done next year. And he said no chance. So that means I know that all of 2024 I won’t have my appointment. I won’t be sitting on my hands.” – Mixed (White and Black African) man, aged 20–29.*

Regarding their operation, patients wanted to know what would occur during the procedure and receive the information needed to make informed decisions about their care (e.g. local or general anaesthetic). Some found the study site’s ‘joint school’ (group information sessions offered to patients prior to their operation) useful, as they got to hear directly from professionals and ask questions in a group setting. Some would have preferred a one-to-one conversation, and others would have liked written communication such as a leaflet. Those who had received a similar operation before, or knew someone else who had, generally felt less anxiety around the operation.

*“I [went] to a seminar with 20 of us with an anaesthetist, a surgeon, a nurse and they tell you what they do and show you the artificial knee thing. And that was interesting, so you know what is happening. And people can ask questions. Especially useful for people who haven’t had one before. A really good idea, yeah it was, knowing what to expect.” – White man, aged 70–79.*

Regarding their recovery, patients wanted to know how much pain they would be in, how long the recovery time would be, and what level of mobility to expect after the operation. Again, those with prior experience of the operation felt less anxiety around the recovery period.

*“Mentally it did [impact me], getting round the fact that you don’t know how long you’re going to be off. Thinking about how it’s going to go. I think I could’ve had more from the doctors. Even though the doctor was good, I know they got a lot to do, but you wanna ask them certain questions, like about the angle, how much [mobility] am I going to get back, but you know you only get a certain amount of time.” – Black man, aged 60–69.*

Patients received emotional support from a variety of sources. Many were encouraged by their friends and family, and this helped them to keep going. Some also found reassurance in their faith and received support from their religious communities.

*“During the time of waiting, my family were very supportive. We even got closer. My girlfriend at the time was super supportive, her friends as well. ‘Don’t worry, they’re going to fix your leg.’ I definitely had a good support system at that time.’” – Mixed (White and Black African) man, aged 20–29.*

When asked about peer support, most patients hadn’t heard of this as an option, and none were offered it by the study site. However, some received informal peer support, either through connections made with other patients at the hospital, or through existing friends or neighbours who had received similar operations. Those who received this informal support found it was helpful to speak with someone who could understand what they were going through and put their situation into perspective.

*“The waiting room, when you are waiting for a taxi, is like an old people’s Gogglebox – they are my peer support. You have a joke and a laugh with them. It’s good to engage and hear people’s stories. If I told my friends about the pain, they would be like, ‘Shut up, mate.’ It’s different when you speak to someone with a life-changing injury. It helps.” – Black Caribbean man, aged 30–39.*

A few patients also said their relationship with their doctors and nurses provided reassurance. This was particularly true for patients who had suffered extensive injuries and had been with the same care team for a long period of time.

*“I’ve known some of these doctors from the start of my accident, six years. So, I know I’m in the right hands. I have a good relationship with most of them. He knows my mind; he knows my family. I’m comfortable.” – Mixed (White and Black African) man, aged 20–29.*

### Co-designing targeted and bespoke support initiatives and identifying how these might help people to live well while waiting

#### Patient demographics

Six patients attended the workshop. All workshop participants lived in an area with the highest level of deprivation (IMD quintile 1). One participant’s preferred language was Farsi, and a Farsi interpreter was present at the workshop. Further detail on the demographics of the patients at the workshop can be found in Table 3.

**Table 3:** Participant characteristics for co-design workshop

*Category*	*n*	*%*
**Ethnicity**		
Black African	1	17
Black Caribbean	1	17
Black – Any other Black	1	17
Mixed – White and Black African	1	17
Mixed – Any other	1	17
Other	1	17
**Age**		
20–29 years old	1	17
40–49 years old	1	17
50–59 years old	1	17
60–69 years old	2	33
70–79 years old	1	17
**Gender**		
Female	4	67
Male	2	33
**Preferred language**		
English	5	83
Farsi	1	17

#### Co-design workshop

At the workshop, participants reflected on the interview insights and shared how these findings resonated with their own experiences. Participants then split into two groups and worked together to design support ideas around the three wellbeing sub-themes: pain, mobility, and hope. From this, participants developed four ideas to support wellbeing.

#### Idea 1: Check-in text, letter, or call

The first idea was to send a text message or letter to patients on the waiting list every three months which acknowledges how long the patient has been waiting and signposts to relevant support (e.g. physiotherapy, peer support groups). Additionally, an administrative staff member would call patients after they had been waiting for a year, or after they had an appointment cancelled, to check in on their wellbeing. Participants felt this would ensure patients do not feel forgotten about, help patients learn about the support services available, and give patients an opportunity to speak directly with the orthopaedics service.

#### Idea 2: Physiotherapy invite letter

The second idea was to send letters written by patients for patients, in which past patients would share first-hand accounts of how physiotherapy helped them while waiting for their procedure and explain the various physiotherapy options offered by the study site. Several interview participants said that seeing physiotherapy work for their peers motivated them to complete their exercises, and workshop participants felt that stories were a powerful way to deliver a message – although they noted the need to be realistic about the potential benefits of physiotherapy.

#### Idea 3: Coping toolkit

The third idea was to create *A window for wellbeing while waiting* – a booklet featuring coping mechanisms recommended by patients to support people while waiting. The booklet would encourage people to consider what they can do to support their wellbeing while waiting, such as picking up a hobby or learning a language, rather than feeling they must put life on hold until after the operation. Participants felt this booklet could help patients feel more empowered and in control while waiting by giving them practical tools to support their physical and mental wellbeing.

#### Idea 4: Patient-centred staff training

The fourth idea was to co-design a patient-centred training for healthcare and administrative staff to help them empathise with the breadth of issues people face while waiting. This could be delivered in a creative way such as a play or film, or through written case studies. Participants felt that positive interactions with NHS staff would improve their overall experience of waiting and wanted staff to consider the role they can play in a patient’s treatment journey, not just medically, but emotionally as well.

## Discussion

### Key findings

This study identified key factors which can impact patients’ wellbeing and quality of life while waiting for an orthopaedic procedure, focusing on those living in more deprived areas. We noted a distinction between the immediate impacts of waiting on patients’ wellbeing, including physical pain, limitations on daily activities, greater reliance on friends and family, and anxiety around the wait; and the longer-term consequences that arise due to extended waits and repeated cancellations, including physical deterioration, delays of life plans, changes in hopefulness, and reduce trust in the NHS. This highlights the heightened and avoidable negative impacts experienced by patients as a result of longer wait times.

Patients with more severe orthopaedic issues or multiple health conditions were more likely to face delays in treatment due to medical complications or the need for additional diagnostic tests. Additionally, patients with caring or financial responsibilities and those with less trust in the NHS due to previous negative experiences were more negatively impacted by their wait than others. These findings contribute to the understanding of why patients in more deprived areas are more likely to wait longer for treatment and are more likely to be negatively impacted by that wait.

We identified four key support needs for patients while waiting: medical support to assist with pain and deterioration, practical support to assist with daily tasks, informational support to ease anxiety while waiting, and emotional support to help patients remain positive. Notably, informational and emotional support were the key areas in which patients wanted more from health professionals. This highlights the importance of patient-health professional interactions as an opportunity to both inform and reassure patients. Several patients also noted the key role that their friends and family played in supporting them. This highlights the need to consider how the health system can provide additional help to those without existing support networks, such as through peer support.

To turn these findings into actionable insights, we worked with patients to co-design four interventions which could support patients to live well while waiting for orthopaedic treatment: check-in messages to let patients know they haven’t been forgotten and signpost them to support offers; a physiotherapy invite letter written by patients for patients to share first-hand accounts of how physiotherapy has helped; a coping toolkit which shared activities that past patients have found helpful to support their wellbeing while waiting; and patient-centred staff training which helps staff empathise with the breadth of issues people face while waiting. These ideas reflect patients’ desire for realistic expectations; candid, empathetic, and consistent communication from healthcare staff; and support offers informed by the experiences of real patients.

### Evidence in context of existing research

Previous literature has identified a lack of research exploring the impacts of waiting for elective surgical care on patients’ wellbeing and quality of life [9, 16]. This study adds to the evidence base and offers suggestions for patient-centred interventions to reduce the identified impacts. Our findings are consistent with existing evidence that long waits for care can result in deteriorating physical, mental, and social wellbeing for patients [4, 8, 17, 20, 24].

One study, which used validated questionnaires for depression and anxiety to explore the relationship the mental health of patients awaiting hip replacement surgery, found no evidence that mental health was worse in those waiting longer for their surgery [3]. Our findings suggest that while extended waits for orthopaedic care may not result in the development of a clinically significant mental disorder, they do lead to notable longer-term consequences which can take a significant toll on patients’ self-reported mental wellbeing and quality of life.

Through our focus on the experiences of patients living in more deprived areas – a group previously identified as facing longer wait times and experiencing worse outcomes while waiting [4, 19, 24] – we were able to provide evidence for a demographic often under-represented in research. This produced insights around challenges which are more common for this group, such as managing caring and financial responsibilities while waiting for treatment.

While health systems often focus on providing patients with prehabilitation and education to improve their fitness, diet, and mental health ahead of surgery [11, 24], our findings are consistent with previous studies which show that patients are more interested in having their concerns acknowledged and addressed by health professionals, and receiving periodic updates about their wait status to manage expectations [4]. When patients did request information about prehabilitation or coping strategies, they preferred this advice came from peers rather than health professionals.

NHS England has released guidance stating that, as of 12 May 2023, any patient waiting for treatment should be contacted every 12 weeks to ask if they still need care and wish to remain on the waiting list [5, 10]. From a provider perspective, this validation can improve the accuracy of waiting lists and reduce waiting times for other patients. From a patient perspective, our findings suggest that it would be beneficial to use these touchpoints as an opportunity to acknowledge and address patient concerns. While digital validation systems are increasingly being used to reduce the administrative burden on hospital staff, providers should also give thought as to how these touchpoints can be used to allow patients to discuss any questions or concerns with a health professional.

### Study strengths

Patient and public involvement was a key strength of this study. Involving public partners throughout the project was critical in ensuring our interview guide was comprehensive, participants felt safe sharing their thoughts and experiences, and the findings accurately reflected what we heard from patients.

Additionally, our study highlighted the experiences of patients living in deprived areas, a group often underrepresented in research. We did this by partnering with the study site’s Business Intelligence team to actively identify and contact eligible patients, rather than taking a passive approach of recruiting through the community. This also allowed us to sample purposively to achieve diversity in terms of ethnicity, gender, age, and reason for procedure. Actions such as offering patients choice over whether their interview took place over the phone, on a video call, or in person; scheduling interviews at a time that was convenient for them; and arranging to have a Farsi interpreter at the workshop also helped to make the study more inclusive. All interview and workshop participants were paid £25 per hour for their time to make the opportunities more accessible.

Finally, the use of a co-design workshop to turn interview insights into patient-centred initiatives made the findings from this study more actionable, and the study site has already secured funding to take forward one of the proposed ideas.

### Study limitations

This study has several limitations. Although effort was made to recruit a representative sample, no Asian patients accepted the invitation to complete an interview. We gave Asian patients who declined an interview the option to share their experiences through an online survey sent to them by the study site via text, but no patients took this up. Further work should be done to understand how to best engage with this group, including acknowledgement of the historical, political, and social context within which they may mistrust research and/or the health system, as well as any unique barriers this group may face in participating in research.

There were relatively high dropout rates for both the interviews and the workshop. Reasons for cancellation included illness, medical appointments, and being in too much pain on the day. This indicates there may have been attrition bias, as patients with more severe health conditions may have been more likely to drop out. The public partners noted that patients living in more deprived areas may be more likely to have competing commitments (e.g. multiple jobs, caring responsibilities) which they would have to re-arrange in order to attend a one-off event like this. Such patients would therefore be less likely to accept an invitation to take part and more likely to drop out. The public partners also noted that those who had experienced discrimination during their care may have less trust in the health system and therefore may be less inclined to take part in this study.

The workshop was held in a ground floor community venue near an underground station and bus stop. However, several patients who were invited to take part said they were not able to due to their limited mobility. In future studies, an online or hybrid option should be offered, particularly when working with patient populations who have limited mobility.

This study was carried out in one clinical specialty, focusing on one specific group (IMD quintile 1), and within one large NHS trust based in London’s city centre. As such, the findings may not be directly transferable to other clinical specialties or demographic groups without further evaluation of their own specific barriers to waiting well. However, we believe that the findings of this work will be transferable to similar clinical settings.

### Opportunities for future work

This was an exploratory study, and the themes generated during our analysis should therefore be viewed as such. It would be of benefit to conduct further qualitative studies with larger sample sizes to explore these themes in more depth, and across a broader range of population subgroups. Further work should also be done to design, test, implement, and evaluate the support ideas generated through this study. This work should be carried out with patients and hospital staff to ensure the acceptability and feasibility of these support interventions. Additionally, there is opportunity to replicate this work with other specialties, as patients’ experiences of waiting and support preferences may vary based on the treatment they are waiting for. Similarly, this work could be replicated with other population groups which experience inequity in wait times, as these groups may have unique support needs.

## Conclusion

Patients are waiting longer for care than ever before. This study highlights the negative impacts that prolonged waits for orthopaedic treatment can have on patients’ physical, mental, and social wellbeing. Waiting has immediate impacts in the form of pain, limited mobility, reduced independence, and anxiety, and patients with caring or financial responsibilities may be disproportionately affected. With extended waits and repeated cancellations, these impacts can be heightened, and patients can experience physical deterioration, delays of life plans, changes in hopefulness, and reduced trust in the NHS. The support needs expressed by patients focused more on acknowledgement of their concerns and management of their expectations by health professionals, rather than advice around lifestyle changes and coping mechanisms. When patients did request such advice, they preferred this information come from peers rather than health professionals. This study offers a number of support ideas designed by patients which can be further developed and implemented by health services to better support patients to live well while waiting for orthopaedic treatment.

## Supplementary Information

Additional file 1

Additional file 2

## Data Availability

The discussion guide and workshop worksheet used in the study are provided as supplementary information files. The data generated or analysed during this study are available from the corresponding author on reasonable request.

## Abbreviations

COVID- 19: Coronavirus Disease 2019
IMD: Index of Multiple Deprivation
NHS: National Health Service
UK: United Kingdom
