# Supplementary material for: Patient experiences of waiting for orthopaedic care and priorities for ‘waiting well’: a qualitative study in a London NHS trust
Source: Arch Public Health. 2025 Apr 7;83:95. doi: 10.1186/s13690-025-01578-4 (PMC11974020; doi:10.1186/s13690-025-01578-4)
Supplement: Supplementary file 1 — Additional file 1 [file 13690_2025_1578_MOESM1_ESM.docx]

**Waiting Well interview discussion guide**

**Aims**

- To understand the challenges faced by people living in more deprived areas while waiting for orthopaedic treatment.
- To identify and co-design targeted initiatives that might help people to live well while waiting for orthopaedic treatment.

**01 Intro and housekeeping**

- Welcome the participant and thank them for taking the time to speak with us.
- Introduce the people in the room and their roles.
- Check that they’re comfortable and offer time to grab water if needed.
- Give background on the Helix Centre, Imperial College Healthcare NHS Trust, and the project.
  - Give context around wider work going on at the Trust to reduce waiting times and address disparities.
  - Make clear that this project is focused on how we can improve the experience while waiting for treatment (rather than the treatment itself or after care).
  - Make clear that we are not clinical staff members and cannot give them medical advice or explain why certain things have happened. This project is about understanding patients’ experiences, but we can signpost them to the Patient Advice and Liaison Service (PALS) for support with their medical care.
- Confirm they have read and signed the consent form and ask whether they have any questions about it.
  - Explain that their data will be stored securely by Imperial College London and deleted within 6 months of the end of the project, and that their responses will be anonymised so that anything said cannot be linked back to their name and will not impact their medical care.
  - Explain that they can ask to take a break or choose to end the interview at any point. They can also choose not to answer a specific question if they don’t feel comfortable doing so.
  - Explain that after the interview, they can request to see their data or withdraw their data by emailing or calling the project lead. However, once reports have been published, it may not be possible to withdraw any data included in the reports.
- Ask if they have any questions before we begin.

**02 Understanding the experience**

**Background**

- To start off, could you tell me roughly how long you have been waiting for your operation?
  - *Show empathy and recognise how difficult this must be to manage.*
- Did you have any expectation of how long you would wait for your treatment? If yes, how long did you expect to wait? And what source did your information on waiting times come from?
- What do you think has impacted your waiting time, if anything?
  - Have you had to move or cancel an appointment? If yes, why was this? Do you think this has impacted your waiting time? How so?
  - Do you have any other health conditions? If yes, do you think this has impacted your waiting time? How so?

**Impact of waiting**

- How has waiting impacted your **physical wellbeing**, if at all?
  - For example, has your condition worsened or are you experiencing any pain?
  - How has this changed over time, if at all?
- How has waiting impacted your **mental wellbeing**, if at all?
  - How has this changed over time, if at all?
  - How has your sense of hope changed over time, if at all?
- How has waiting impacted your ability to carry out **daily tasks**, if at all?
  - How has this changed over time, if at all?
  - Has a partner of family member supported you with this? If yes, how has this impacted them? How has this impacted your relationship with them?
  - How has your self-worth or sense of value changed over time, if at all?
- How has waiting impacted your relationships with **family and friends**, if at all?
  - How has this changed over time, if at all?
- Do you have any other comments on the impact that waiting had?
- Has your view of the NHS changed over the last 6 months? If yes, how so?

**Coping strategies**

- We want to chat about your time spent while waiting.
  - Have you explored or used any **medical interventions** (e.g. painkillers, physiotherapy, psychological therapy)? If yes, what are they?
  - Have you explored or used any **alternative or traditional methods** (e.g. traditional medicine, religious practices)? If yes, what are they?
  - Have you made any **lifestyle changes** to help prepare for your procedure (e.g. diet, exercise)? If yes, what are they?
  - Do you think that waiting has led you to take up any **unsafe or unhelpful coping strategies**? If yes, do you mind sharing more about this?
    - *N.B. Responses may highlight a need for signposting to further support.*
- How have these coping strategies impacted your physical and mental wellbeing, if at all?

**Support**

- What support have you received while waiting?
  - How have your **family and friends** supported you, if at all?
  - How have **health professionals** supported you, if at all?
  - Have you explored or used any **peer support** services (i.e. support from other people with similar experiences or waiting for similar treatment)?
- How has this support impacted your physical and mental wellbeing, if at all?

**Communication**

- What communication have you had from the **orthopaedics service** while waiting, if any?
- What communication have you had from your **GP** while waiting, if any?
- How do you feel about the level of communication you have received while waiting?
- Have you contacted the service yourself for information about your wait or appointment?
  - If yes, why did you contact the service? How was your experience?
  - If no, do you feel able to contact the service yourself? Why or why not?

**03 Understanding what good looks like**

- How would you improve the current experience of waiting?
  - What communication would be helpful while waiting?
  - What advice would be helpful while waiting?
  - What support would be helpful while waiting?
  - Is there any advice or support you have found helpful, and think should be made more widely available?

**04 Wrap up**

- Ask if there is anything else they would like to tell us.
- Thank them for their time and for sharing their experiences with us.
- Explain that we will use the insights from these interviews to inform a workshop where we will work with patients to co-design tailored support interventions. The findings from these interviews and the workshop will be shared with Imperial College Healthcare NHS Trust, and the recommendations will help to inform their next steps. We will share a summary of the key learnings and recommendations with participants at the end of the project.
- Offer a debrief and signpost to the support organisations listed on the consent form in case anything discussed brought up difficult thoughts or feelings.
- Ask whether they would like to be paid via bank transfer or gift voucher and sort out the logistics (i.e. NPF form for bank transfer, shop preference for gift voucher).
- Ask if they have any questions. Remind them that they can contact Jodie if they think of any questions after the interview.
- Thank them again for their time.
