# Supplementary material for: Patient experiences of waiting for orthopaedic care and priorities for ‘waiting well’: a qualitative study in a London NHS trust
Source: Arch Public Health. 2025 Apr 7;83:95. doi: 10.1186/s13690-025-01578-4 (PMC11974020; doi:10.1186/s13690-025-01578-4)
Supplement: Supplementary file 2 — Additional file 2 [file 13690_2025_1578_MOESM2_ESM.pdf]

Idea name:

Our idea is to...

We think this will support patients to wait well because...

This idea would work well for someone who...

This idea might not work for someone who...

We would let patients know this support exists by...

We would need these things to make our idea a reality...

A drawing of the idea
